# Educational technologies for families and children with type 1 diabetes: a scoping review*

**DOI:** 10.1590/1980-220X-REEUSP-2024-0134en

**Published:** 2025-01-13

**Authors:** Patricia Carli Morgado, Liliane Faria da Silva, Rosane Cordeiro Burla de Aguiar, Juliana Rezende Montenegro Medeiros de Moraes, Tatiane Marinz de Souza Luquez, Euzeli da Silva Brandão, Michelle Darezzo Rodrigues Nunes, Isabelle de Freitas Lopes, Débora Câmara de Campos

**Affiliations:** 1 Universidade Federal Fluminense, Escola de Enfermagem Aurora de Afonso Costa, Mestrado Profissional em Enfermagem Assistencial, Niterói, RJ, Brazil.; 2 Universidade Federal do Rio de Janeiro, Escola de Enfermagem Anna Nery, Programa de Pós-Graduação Stricto Sensu, Rio de Janeiro, RJ, Brazil.; 3 Universidade Federal Fluminense, Escola de Enfermagem Aurora de Afonso Costa, Departamento de Enfermagem Materno-Infantil e Psiquiátrica, Niterói, RJ, Brazil.; 4 Universidade do Estado do Rio de Janeiro, Faculdade de Enfermagem, Departamento de Enfermagem Materno-Infantil, Rio de Janeiro, RJ, Brasil.; 5 Universidade Federal do Rio de Janeiro, Instituto de Puericultura e Pediatria Martagão Gesteira, Rio de Janeiro, RJ, Brazil.

**Keywords:** Diabetes Mellitus, Type 1, Educational Technology, Child, Caregivers, Health Education, Diabetes Mellitus Tipo 1, Tecnología Educacional, Niño, Cuidadores, Educación en Salud

## Abstract

**Objective:**

To map scientific evidence on educational technologies developed for family members and children with type 1 diabetes.

**Method:**

Scoping review, according to JBI recommendations, and described in accordance with the checklist PRISMA-ScR. Searches were carried out in the LILACS, BDENF, PUBMED, COCHRANE, CINAHL, EBSCO, Scopus and Embase/Elsevier, Web of Science/Clarivate Analytics, Scielo, VHL Regional Portal and gray literature databases.

**Results:**

Fifty-three studies published between 1980 and 2023 were included. The evidence was categorized into digital educational technologies, which provide innovative resources to educate and support children and families, and non-digital educational technologies, which provide practical and interactive opportunities for learning about diabetes.

**Conclusion:**

The results highlight the relevance of educational technologies in the care of children with type 1 diabetes. However, they reveal a gap in the assessment of the effectiveness of these interventions in the long term, with regard to adherence to treatment and improvement in quality of life. Research is required to evaluate the effectiveness of these technologies and the impact of educational interventions.

## RESUMEN

**Objetivo:**

Mapear evidencia científica sobre tecnologías educativas desarrolladas para familias y niños con diabetes tipo 1.

**Método:**

Revisión del alcance, según recomendaciones del JBI, y descrita de acuerdo con el lista de verificación PRISMA-ScR. Las búsquedas se realizaron en las bases de datos LILACS, BDENF, PUBMED, COCHRANE, CINAHL, EBSCO, Scopus y Embase/Elsevier, Web of Science/Clarivate Analytics, Scielo, Portal Regional de la BVS y literatura gris.

**Resultados:**

Se incluyeron 53 estudios publicados entre 1980 y 2023. La evidencia se clasificó en tecnologías educativas digitales, que ofrecen recursos innovadores para educar y apoyar a los niños y las familias, y tecnologías educativas no digitales, que brindan oportunidades prácticas e interactivas para aprender sobre la diabetes.

**Conclusión:**

Los resultados resaltan la relevancia de las tecnologías educativas en el cuidado de niños con diabetes tipo 1. Sin embargo, revelan una brecha en la evaluación de la efectividad a largo plazo de estas intervenciones, en lo que respecta a la adherencia al tratamiento y la mejora de la calidad de vida. Se necesita investigación para evaluar la efectividad de estas tecnologías y el impacto de las intervenciones educativas.

## INTRODUCTION

Type 1 Diabetes Mellitus (DM1) is the second most prevalent chronic disease in childhood, with an average of 600,000 cases among children and adolescents aged 0 to 14 years. Brazil is the third country with the highest number of children and adolescents with type 1 diabetes in the world, with a prevalence of approximately 51 thousand cases in the age group from 0 to 14 years old^(1)^.

DM1 is caused by an autoimmune process where the immune system attacks the pancreatic beta cells, resulting in decreased or absent insulin production^(1)^. It is considered one of the most common and serious chronic childhood diseases, due to the complications it can cause in the short, medium, and long term, such as damage to blood vessels, the heart and brain, retinopathy, neuropathy, nephropathy and diabetic ketoacidosis, among which kidney disease is the most frequent and earliest found in young people with diabetes^(1,2)^.

Chronic illness in childhood limits daily activities, affects the growth and development process, requires ongoing health care, and affects the daily lives of all family members. Therefore, family members who assume responsibility for care need to acquire knowledge about nutrition, signs and symptoms of hypoglycemia and hyperglycemia, in addition to being trained in the preparation and administration of insulin^(3)^.

Health education is an important structure in the care of children with diabetes, promoting actions to improve and make care provided in the home environment more effective for children with this chronic disease^(4)^.

In the context of DM1, different educational technologies (ET) can be incorporated to support the educational actions of health professionals, facilitate learning, and promote adherence to treatment and self-care^(5)^.

Therefore, a preliminary search was carried out in July 2023 at PROSPERO (*International Prospective Register of Systematic Reviews*) and in the main databases and no current or ongoing scoping or systematic review on the subject was identified.

Considering the relevance of the topic, this review aims to map, through scientific productions, educational technologies for family members and children with type 1 diabetes mellitus.

It is expected that this scoping review will provide support for new studies on the topic in question, to encourage the participation of family members and children through educational technologies in coping DM1.

## METHOD

### Design of Study

This is a scoping review, conducted in accordance with JBI recommendations^(6)^ and described in accordance with the checklist *Preferred Reporting Items for Systematic Reviews and Meta-Analyses Extension for Scoping Reviews* (PRISMA-ScR)^(7)^. In the exploratory phase of this review, we aimed to ensure the absence of a recent research report similar to the topic under study or record of a review protocol. Subsequently, the protocol of this review was registered on the platform *Open Science Framework* (https://doi.org/10.17605/OSF.IO/A72F4).

### Identifying the Research Question

Review driven by the following question: “What are the educational technologies for families and children with type 1 diabetes? To construct the research question, the mnemonic strategy PCC (Population, Concept and Context) was used, considering as population (P) – children with type 1 diabetes and/or family members; the concept (C) – educational technologies; and the context (C) – health care environments.

The inclusion criteria were studies whose population consisted of children aged 2 to 12 years and adolescents, and the exclusion criteria were studies whose population was exclusively composed of adolescents, studies that specifically deal with diabetic ketoacidosis and studies aimed exclusively at teachers or health professionals.

### Selection Criteria

Experimental and quasi-experimental studies (randomized and non-randomized controlled studies, before-and-after studies, and interrupted time series studies), analytical observational studies (prospective and retrospective cohort studies, case-control studies and cross-sectional analytical studies), descriptive observational studies (case series, individual case reports and descriptive cross-sectional studies), and qualitative studies were considered. Systematic reviews that met the inclusion criteria were also considered for analysis and selection of their reference lists.

### Search Strategy

The search was carried out in the LILACS, BDENF, Coleciona SUS databases, among others from the BVS Regional Portal, MEDLINE via Pubmed/NLM, Pubmed Central/NLM, COCHRANE Library, CINAHL, EBSCO, Scopus and Embase/Elsevier, Web of Science/Clarivate Analytics, Scielo, according to the syntax and indexing terms appropriate for each database. Likewise, Google, Google Scholar and grey literature sources such as Science.gov and USA.gov were considered. The search strategy is listed in Chart 1. References from all included sources of evidence were screened for additional studies. To expand on the findings, the reviewers defined the strategy with the help of a librarian.

**Chart 1:** Search strategy used in the review – Niterói, RJ, Brazil, 2023.

((“Diabetes Mellitus, Type 1”[mh] OR “Diabetes Mellitus, Type 1”[tiab] OR “Autoimmune Diabetes”[tiab] OR “Brittle Diabetes Mellitus”[tiab] OR IDDM[tiab] OR “Insulin Dependent Diabetes Mellitus 1”[tiab] OR “Insulin-Dependent Diabetes Mellitus”[tiab] OR “Insulin-Dependent Diabetes Mellitus 1”[tiab] OR “Juvenile Onset Diabetes”[tiab] OR “Juvenile-Onset Diabetes”[tiab] OR “Juvenile-Onset Diabetes Mellitus”[tiab] OR “Ketosis-Prone Diabetes Mellitus”[tiab] OR “Sudden-Onset Diabetes Mellitus”[tiab] OR “Type 1 Diabetes”[tiab] OR Type 1 Diabetes Mellitus[tiab]) AND (“Health Education”[mh] OR “Health Education”[tiab] OR Educac*[tiab] OR “Educational Technology”[mh] OR Educational Technolog*[tiab] OR Instructional Technolog*[tiab] OR “health fairs”[tiab] OR health science* education[tiab] OR Pamphlets[mh] OR Booklet*[tiab] OR Brochure*[tiab] OR Pamphlet* Instructional[tiab] OR Instructional Film[tiab] OR Instructional Video[tiab] OR Instruction[tiab] OR Audiovisual Demonstration[tiab] OR Audio-Video Demonstration[tiab] OR Audiovisual Demonstration[tiab] OR Instruction[tiab] OR Instructional Films[tiab] OR Instructional Videos[tiab] OR Video-Audio Demonstration[tiab] OR “Audiovisual Aids”[mh] OR Audio Visual Aid*[tiab] OR Audio-Visual Aid*[tiab] OR Audiovisual Aid[tiab] OR Visual Aid*[tiab] OR “Teaching Materials”[mh] OR “Teaching Material”[tiab] OR booklet[tiab])) AND (“Infant”[mh] OR Infant*[tiab] OR “Child, Preschool”[mh] OR Preschool*[tiab] OR Pediatric*[tiab] OR “Child”[mh] OR Child*[tiab] OR Children[tiab] OR Non-Professional Home Care[tiab] OR Nonprofessional Home Care[tiab] OR Housing[mh] OR Family Patient Lodging[tiab] OR Family-Patient Lodging*[tiab] OR Patient Family Lodging*[tiab] OR Patient-Family Lodging*[tiab] OR “Home Environment”[mh] OR Home Environment*[tiab] OR Living Alone[tiab] OR Social Housing Condition*[tiab] OR “Foster Home Care”[mh] OR Adult Foster Care[tiab] OR Fostering[tiab] OR Kinship Care[tiab] OR “Caregivers”[mh] OR Caregiver*[tiab] OR Carer*[tiab] OR “Care Givers”[tiab] OR “Care Giver”[tiab] OR family care*[tiab] OR “unpaid care”[tiab] OR informal care*[tiab] OR “Family”[mh] OR Families[tiab] OR Filiation[tiab] OR relatives[tiab] OR Stepfamil*[tiab] OR Parent*[tiab] OR “Step Parents”[tiab] OR Step-Parent[tiab] OR Step-Parent*[tiab] OR Stepparent*[tiab] OR maternity[tiab] OR motherhood[tiab] OR parenthood[tiab] OR paternity[tiab] OR “mothers”[mh] OR “Fathers”[mh] OR “mothers”[tiab] OR”Fathers”[tiab])

### Study Selection

For the initial stages of study selection, the Rayyan QCRI® platform (the Systematic Reviews web app) was used. Two independent reviewers verified the relevance of the selected studies by reading the titles and abstracts. Disagreements regarding the inclusion of articles were resolved through discussion among the reviewers, without the need for intervention by a third reviewer. Subsequently, the selected articles were read in full, which preceded their inclusion in the final sample.

#### Data Extraction and Synthesis

To systematize the results, the extracted data were compiled, in a descriptive manner, in a chart developed by the authors and adapted to the recommendations of the JBI manual^(6)^. The following information was highlighted: author, year, country, type of study, participants, type and content of technologies.

## RESULTS

**Figure 1:**
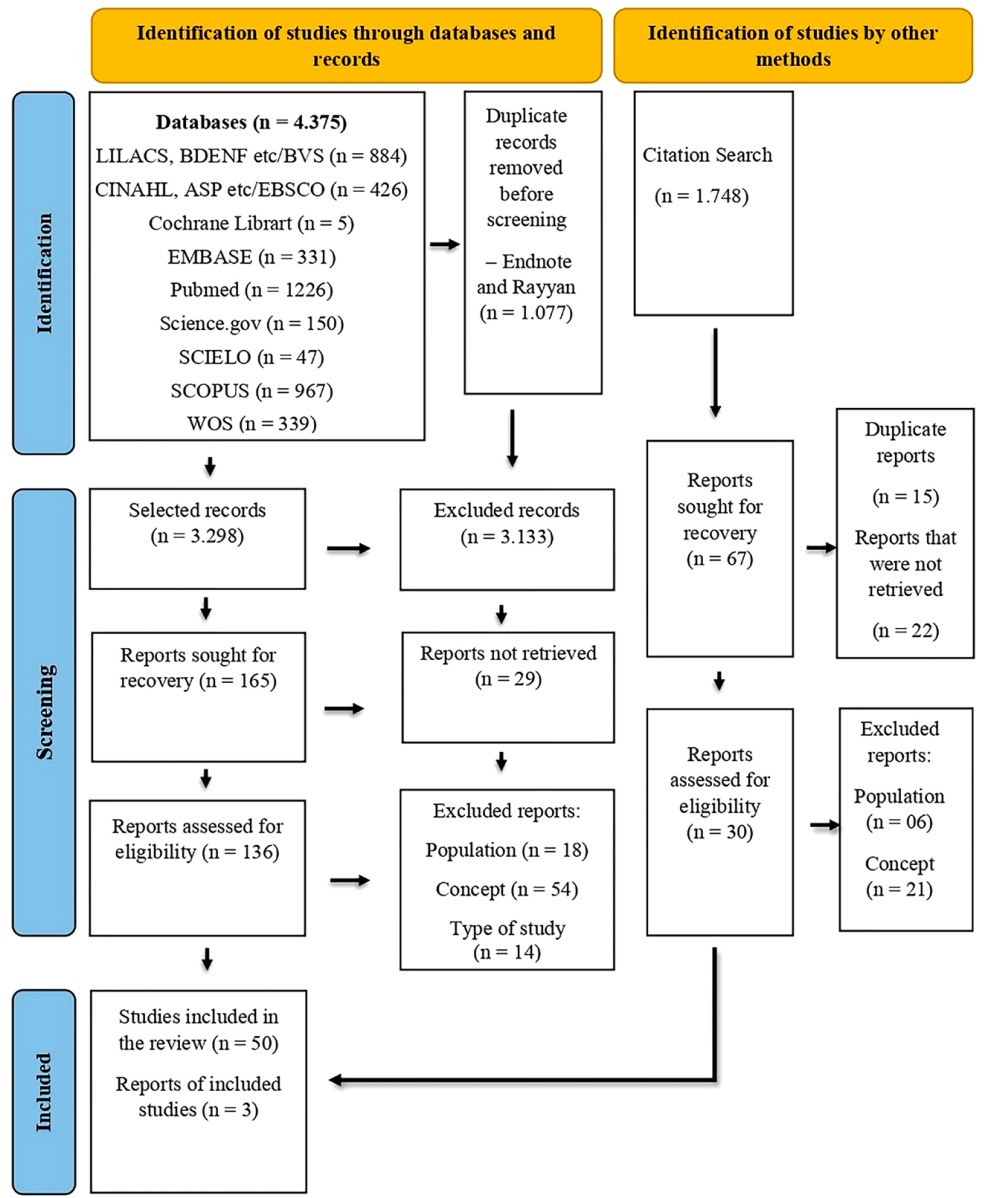
PRISMA-ScR flow diagram of the review publication selection process. Niterói, RJ, Brazil, 2023.

The database search took place from September to December 2023. The search strategy used resulted in 4375 records, of which, after removing duplicates, 3296 articles were included for initial screening and analysis of titles and abstracts. From this stage, 165 articles were selected for full reading. Among those selected, 86 were excluded because they did not fully address the topic and 29 could not be recovered, totaling 50 articles included. Furthermore, after analyzing the references, 3 more studies were added, composing a final sample of 53 studies selected for the review (Figure 1).

Most articles were published between 2014 and 2020. Regarding the origin of the studies, Brazil was the country with the largest scientific production on the subject, accounting for 17 articles, followed by the United States, with 16 articles. The largest concentration of studies was experimental trials (13 studies) (Chart 2).

**Chart 2:** Characterization of the studies included in the final review sample – Niterói, RJ, Brazil, 2023.

Code	Author Year Country	Design of study	Participants	Educational Technologies
A1^(8)^	Sunni M et al. 2023 USA	Cross-sectional study	22 Somali parents of 22 children	Educational video
A2^(9)^	Merino MFGL et al. 2022 Brazil	Qualitative, convergent-care study	16 children/teenagers	Educational activities: toys, games, videos and printed materials
A3^(10)^	Ribeiro ALT et al. 2021 Brazil	Methodological study	16 Children/teenagers	Comics
A4^(11)^	Sparapani VC et al. 2021 Brazil	Methodological study	19 children	Computer-assisted instruction program
A5^(12)^	Araujo et al. 2020 Brazil	Methodological study	16 Children/teenagers	Comics
A6^(13)^	La Banca RO et al. 2020 Brazil	Descriptive qualitative study	20 children/teenagers	Instructional Therapeutic Toy
A7^(14)^	Chaves FF et al. 2020 Brazil	Methodological study	10 children/teenagers	Protocol for App
A8^(15)^	La Banca RO et al. 2020 Brazil	Randomized pilot trial	20 children	Therapeutic Play Intervention (PTI)
A9^(16)^	La Banca RO et al. 2019 Brazil	Integrated qualitative case study	2 Children	Instructional therapeutic toy
A10^(17)^	Serafim AR et al. 2019 Brazil	Methodological study	16 children/teenagers	Serious games
A11^(18)^	Albanese-O’Neill A et al. 2019 USA	Mixed methods study	30 Parents of children with DM1	Site
A12^(19)^	Rafeezadeh E et al. 2019 Iran	Randomized Clinical Trial	68 children	Interactive educational video game
A13^(20)^	Marker AM et al. 2019 USA	Experimental studies	14 parents	Online platform: micro-video lectures
A14^(21^)	Kyfonidis C, Lennon M 2019 Scotland	Experimental study	27 parents and 21 children	Interactive activity with tangible educational toys
A15^(22)^	Emiliana P et al. 2019 Indonesia	Quasi-experimental study without control group	31 children	Animated videos
A16^(23)^	Pennafort VPS et al. 2018 Brazil	Qualitative study	26 children	Therapeutic toy in cultural care
A17^(24)^	Kaneto LA et al. 2018 Brazil	A quasi-experimental study	33 children	Educational workshop/games
A18^(25)^	Bernier A et al. 2018 USA	Randomized Clinical Trial	16 children	Educational application
A19^(26)^	Henkemans OAB et al. 2017 Netherlands	Randomized Clinical Trial	27 children/teenagers	Personal robot
A20^(27)^	Moura DJM et al. 2017 Brazil	Methodological Research	19 children aged 8 to 11	Educational booklet
A21^(28)^	McCulloch V et al. 2017 United Kingdom	Experimental study	5 children/teenagers	Educational and interactive mobile application
A22^(29)^	Bechara GM et al. 2017 Brazil/Belgium	Experimental study	9,944 students, 26 of whom have DM1; 236 teachers and 32 family members	Kids Program (online information package)
A23^(30)^	Mauri A et al. 2017 Italy	Methodological study	24 children	Therapeutic education program (educational workshops)
A24^(31)^	Nilsson S 2016 Sweden	Descriptive qualitative study	15 children	Tablet: human anatomy app, diabetes game, carb counter.
A25^(32)^	Joubert M et al. 2016 France	Prospective multicenter pilot study	38 children	Serious games
A26^(33)^	Ramchandani N et al. 2016 USA	Descriptive qualitative study	31 mothers and 18 fathers	Human Patient Simulator
A27^(34)^	Sullivan-Bolyai S et al. 2016 USA	Randomized Clinical Trial	22 parents and 22 children	Human Patient Simulator
A28^(35)^	Queiroz MVO et al. 2016 Brazil	Descriptive and analytical study	6 children	Dynamics of Body Knowledge and Drawings
A29^(36)^	Chomutare T et al. 2016 Norway	Participatory design	14 children	Co-design or participatory design of serious games on mobile devices
A30^(37)^	Price KJ et al. 2015 United Kingdom	Randomized Clinical Trial	396 children/teenagers	Diabetes Education Course
A31^(38)^	Maguire LL et al. 2015 USA	Randomized Clinical Trial	30 grandparents of children with diabetes	Human Patient Simulator
A32^(39)^	Bin-Abbas B et al. 2014 Saudi Arabia	Prospective experimental trial	200 children	Interactive, informative and multimedia messaging system
A33^(40)^	Narae Kang et al. 2014 Korea	A quasi-experimental study	15 children/teenagers	Lecture, quiz and games
A34^(41)^	Jaser SS et al. 2014 USA	Multicenter Clinical Trial	320 children/teenagers	Online psychoeducational program - coping skills and self-care
A35^(42)^	Grey M et al. 2013 USA	Multicenter Clinical Trial	320 children/teenagers	Internet-based psychoeducational program - comic strip video and links to problem-solving exercises
A36^(43)^	Hanberger L et al. 2013 Sweden	Randomized Clinical Trial	487 (289 children/adolescents and 198 parents)	Web portal
*A37^(44)^	Ramchandani N et al. 2016 USA	Descriptive qualitative study	Parents of children > 13 years (Does not describe the number of participants)	Human Patient Simulator
A38^(45)^	Nicholas DB et al. 2012 Canada	Experimental study with mixed methods	74 children/teenagers	Site
A39^(46)^	Sullivan-Bolyai S et al. 2012 USA	A focus group study and two pilot studies	16 parents	Human Patient Simulator
A40^(47)^	Fuchslocher A, Niesenhaus J, Krämer N 2011 Germany	Intersubject experimental study with clinical sample	20 children	Digital game
A41^(48)^	Souza LR et al. 2010 Brazil	Descriptive qualitative study	10 children and family	Educational brochure
A42^(49)^	Martin C, Liveley K, Whitehead K 2009 United Kingdom	Experimental study	5 children	Group session: storytelling and drawings afterwards / Individual session: app and games
A43^(50)^	Wangberg SC, Årsand E, Andersson N 2006 Norway	Clinical Trial	15 children	Text messaging system – SMS
A44^(51)^	Pélicand J et al. 2006 France	Quasi-experimental study with control group	14 children	Therapeutic education program (puppet making; card games; reading; dramatization.
A45^(52)^	Noriaki Aoki et al. 2005 USA	Experimental study	30 children/teenagers	Game App
A46^(53)^	Nordfeldt S et al. 2005 Sweden	Randomized study	332 children/teenagers	Self-study material in videos
A47^(54)^	Noriaki Aoki et al. 2004 Japan	Clinical Trial	58 children	Video game
A48^(55)^	Nordfeldt S et al. 2003 Sweden	Randomized study	332 children/teenagers	Videotapes and a booklet
A49^(56)^	Nordfeldt S, Ludvigsson J 2002 Sweden	Open cohort design	139 children/teenagers	Self-study handouts and video programs
A50^(57)^	Brown SJ et al. 1997 USA	Randomized Clinical Trial	31 children/teenagers	Video game
A51^(58)^	Engvall JC 1994 USA	Descriptive Study	14 children	Video game
*A52^(59)^	Rogari GL, Uster M 1988 USA	Experimental study	Children with diabetes (Does not describe the number of participants)	Educational program: lectures, slides, blackboard, games, dramatization.
A53^(60)^	James VH, Susan JL 1980 USA	Experimental study	37 children/teenagers	Game App

*The articles do not describe the number of participants.

### Categorization of Educational Technologies

Educational technologies were organized into two categories: digital and non-digital educational technologies, comprising the most relevant data found in this study. Digital ETs were websites, platforms, videos, video games, applications, digital games, human patient simulators and text messages (via cell phone). The non-digital ones were booklets, leaflets, books, comics, therapeutic toys, lectures, quizzes, puppets, workshops, groups, and educational programs. The central themes explored in the ET addressed insulin therapy, glycemic monitoring, hyperglycemia, hypoglycemia, physical activity, nutrition, pathophysiology of diabetes, late complications, school and technologies for diabetes.

### Digital Educational Technologies

37 articles were found that address digital technologies^(8,11,14,17-22,25,26,28,29,31-34,36-39,41-47,50,52-58,60)^.

Regarding the videos^(8,10,22,53,55,56)^, these provide an accessible approach, with good cultural acceptance and effective in disseminating information. As for video games^(11,19,32,36,47,54,57)^, they are presented as tools with an innovative approach for children with DM.

Mobile applications and serious games^(14,17,25,28,31,52)^ are useful as they provide resources such as support messages, glycemic monitoring and emotional recording, providing training in the player’s operational and behavioral skills.

Some studies highlight the potential of mobile technologies, such as mobile messaging^(18,39,50)^ and telemedical educational programs^(43.45)^, to provide accessible diabetes education outside of the clinical setting. The use of human patient simulators^(21,26,33,34,38,44,46)^, home educational materials, and computer-assisted teaching programs^(29,37,41,42,53,55,56,58)^ was also highlighted.

### Non-Digital Educational Technologies

In this category, 18 articles were included^(9,10,12,13,15,16,23,24,27,30,35,40,48,49,51,55,56,59)^ that address non-digital technologies. Among these, 3 studies^(9,55–56)^ have adopted approaches that combine both digital and non-digital technologies to achieve their educational goals.

Therapeutic toys^(13,15,16,21,23)^ were used, as well as printed materials (booklets, pamphlets, books, comics)^(10,12,27,48,55,56)^, educational programs implemented both in specialized diabetes camps^(40,49,51)^ and in a hospital outpatient setting^(59)^, in which a variety of activities were conducted, including storytelling, use of puppets, games, dramatizations, lectures and quizzes. Dynamics and workshops were also proposed^(24,35)^, as well as specific strategies to address each difficulty reported by both the child and the mother, with personalized activities adapted to the level of understanding of each dyad^(9)^.

## DISCUSSION

Digital ETs play a crucial role in the management of DM1 by providing culturally sensitive and personalized resources to educate patients and their families. This includes the use of video games, mobile applications, simulators and online interventions, which aim to promote knowledge about DM1, encourage self-care, and improve patients’ quality of life. The use of technological resources, which are more attractive and focused on reality, can facilitate training, stimulate reasoning and the ability to solve problems^(61)^.

Videos, cited by 5 studies in this review, are accessible technologies that can be edited and adapted to cultural diversity and are effective in disseminating information. They can be translated into different languages, dubbed or subtitled, and so are used with different audiences, and also easily distributed to patients through websites, social media, text messages. Also, LIBRAS interpreters may be included and thus the hearing impaired public. Educational videos are an effective strategy, as they capture the public’s attention, stimulate creativity and arouse curiosity about the topic, facilitating educational practice in a direct and simple way^(62)^.

Video games, which were also technologies found in the review, present themselves as innovative educational tools. These games are developed based on behavioral theories and encourage children’s active participation, promoting knowledge about the disease and self-care behaviors in a playful way. Video games provide cognitive benefits, such as attention focus, problem-solving skills, and stimulating creativity. They also have positive repercussions on motivation, mood and positive feelings. Furthermore, they favor social behaviors, such as cooperation and commitment^(63)^.

Mobile apps were identified as important educational technologies, as they offer features such as supportive messaging, blood glucose monitoring, and emotional recording. This way, they complement the self-management of DM1 in children and adolescents, in a combination of educational information and practical tools to control the disease more efficiently. These findings highlight the effectiveness of mobile technologies in empowering parents and supporting the care of children with DM1, encouraging self-care, better glycemic control, physical activity, adoption of healthy eating, and maintenance of prescribed medication^(64)^.

Mobile technologies, such as mobile messaging and telemedical educational programs showed to be able to provide accessible diabetes education outside of the clinical setting. Studies indicate that teleconsultation and telecare allow the monitoring of pediatric patients, enabling assessment and supervision in their usual environments, such as home, school and community. This approach improves access to and quality of health care^(65)^.

Another educational technology described in the literature is human patient simulators (HPS). These simulators are technological devices designed to mimic real clinical situations, allowing caregivers to practice DM1 management skills in a simulated environment. Realistic simulation has emerged as a more effective approach to teaching and learning than traditional teaching. This strategy provides a practical experience closer to reality, contributing to safer and risk-free care^(66)^.

New technologies, such as the use of the Internet to share educational materials, online communications for DM1, and computer-assisted programs, emerged in the review as promising tools to improve care. In this regard, studies on online health platforms and chronic disease management indicate that virtual interactions contribute to greater patient knowledge of the disease, through the sharing of experiences and information; better self-management of the chronic condition, due to the exchange of experiences and advice; and greater social support, including positive reinforcement and sharing of information and experiences^(67)^.

Regarding non-digital technologies, therapeutic toys aimed at children living with DM1 emerged as a playful-therapeutic intervention that promotes the exchange of experiences between the child and the health professional, as well as between peers during group activities. As benefits, it expands knowledge about the disease and facilitates practical training in essential skills for diabetes care, such as blood glucose monitoring and insulin administration. The results obtained in this review show effectiveness in promoting self-care among children.

Games and toys play a significant therapeutic role, encouraging children to express their feelings and anxieties regarding their health condition, helping them to alleviate the tension imposed by their reality. Furthermore, these activities promote greater child-professional interaction^(68)^.

The various types of printed educational materials found in the review results are used as resources for self-study and complement the guidance provided by health professionals. According to studies, printed materials, such as booklets, are resources that remain available for consultation by users and their families when they have questions, facilitating the process of autonomy, and greater adherence to treatment and understanding of the influence of their own actions on their health pattern^(69)^.

Educational programs, with recreational activities and lectures, developed in camps and outpatient clinics were identified as possibilities for action with the study population. These interactive and recreational methods provide children with an opportunity to express and share their diabetes-related difficulties, while also supporting psychosocial skills. As evidenced in a systematic review of educational strategies used in teaching insulin therapy to children and adolescents with DM1, educational programs must prioritize not only strategies that take into account the needs and particularities of the target population, but also ensure ongoing support. This is essential for developing and maintaining self-care behaviors, achieving better results over time^(70)^.

The central themes explored in the ETs presented in this review show the need for an integrated approach that encompasses not only insulin therapy and glycemic monitoring, but also nutrition, physical activity, and coping skills. These are topics consistent with the recommendations of the *American Diabetes Association* (ADA), which emphasizes the importance of comprehensive and ongoing education to optimize health outcomes in patients with diabetes^(2)^, providing a solid foundation to effectively guide children with DM1 and their families in self-care and in the promotion of a better quality of life.

This review identified as a potential limitation the exclusion of 29 articles not retrieved in full, which may generate a bias in the review, as these studies could contain relevant data that were not considered.

Regarding the implications for nursing practice, this review provides a synthesis of educational technologies and topics addressed in the care of children with diabetes described in the literature, which can be replicated, adapted, and used by nurses in their health education interventions aimed at children with diabetes and their families. This analysis supports effective management of diabetes and promotes improved quality of life for these patients and their families.

## CONCLUSION

This scoping review highlighted the diversity and importance of educational technologies in the management of DM1 in children, revealing a range of digital and non-digital tools used to promote self-care and support families. The studies analyzed indicated that these technologies not only increase knowledge about the disease, but also contribute to the development of practical and behavioral skills necessary for the effective management of DM1.

However, several gaps were identified in the literature. Many studies have focused mainly on describing the development of educational technologies, without delving into the analysis of the thematic content covered. Furthermore, the scientific dissemination of these technologies is still limited, restricting their access to a wider audience. There was also a lack of studies evaluating the long-term effectiveness of these technologies and their impact on treatment adherence and the quality of life of children and their families.

Therefore, there is a clear need for future studies that evaluate the effectiveness of educational technologies over time, considering different cultural and socioeconomic contexts; investigate the implementation of these technologies on a large scale, aiming at their integration into public health systems; and explore the thematic content of educational interventions in greater depth, providing specific guidelines for health professionals.

These future investigations can help to consolidate existing evidence and expand the use of educational technologies, promoting more effective and comprehensive care for children with type 1 diabetes and their families.
