# Supplementary material for: Educational technologies for families and children with type 1 diabetes: a scoping review
Source: Rev Esc Enferm USP. 2025 Jan 13;58:e20240134. doi: 10.1590/1980-220X-REEUSP-2024-0134en (PMC11774519; doi:10.1590/1980-220X-REEUSP-2024-0134en)
Supplement: Translation

# Translation: Tecnologias educacionais para familiares e crianças com diabetes tipo 1: revisão de escopo*

**DOI:** 10.1590/1980-220X-REEUSP-2024-0134pt

**Authors:** Patricia Carli Morgado, Liliane Faria da Silva, Rosane Cordeiro Burla de Aguiar, Juliana Rezende Montenegro Medeiros de Moraes, Tatiane Marinz de Souza Luquez, Euzeli da Silva Brandão, Michelle Darezzo Rodrigues Nunes, Isabelle de Freitas Lopes, Débora Câmara de Campos

**Affiliations:** 1 Universidade Federal Fluminense, Escola de Enfermagem Aurora de Afonso Costa, Mestrado Profissional em Enfermagem Assistencial, Niterói, RJ, Brasil.; 2 Universidade Federal do Rio de Janeiro, Escola de Enfermagem Anna Nery, Programa de Pós-Graduação Stricto Sensu, Rio de Janeiro, RJ, Brasil.; 3 Universidade Federal Fluminense, Escola de Enfermagem Aurora de Afonso Costa, Departamento de Enfermagem Materno-Infantil e Psiquiátrica, Niterói, RJ, Brasil.; 4 Universidade do Estado do Rio de Janeiro, Faculdade de Enfermagem, Departamento de Enfermagem Materno-Infantil, Rio de Janeiro, RJ, Brasil.; 5 Universidade Federal do Rio de Janeiro, Instituto de Puericultura e Pediatria Martagão Gesteira, Rio de Janeiro, RJ, Brasil.

**Keywords:** Diabetes Mellitus Tipo 1, Tecnologia Educacional, Criança, Cuidadores, Educação em Saúde

## Abstract

**Objetivo:**

Mapear as evidências científicas sobre tecnologias educacionais desenvolvidas para familiares e crianças com diabetes tipo 1.

**Método:**

Revisão de escopo, segundo as recomendações do JBI, e descrita de acordo com o *checklist* PRISMA-ScR. As buscas foram realizadas nas bases LILACS, BDENF, PUBMED, COCHRANE, CINAHL, EBSCO, Scopus e Embase/Elsevier, Web of Science/Clarivate Analytics, Scielo, Portal Regional da BVS e literatura cinzenta.

**Resultados:**

Foram incluídos 53 estudos publicados entre 1980 e 2023. As evidências foram categorizadas em tecnologias educacionais digitais, que oferecem recursos inovadores para educar e apoiar crianças e familiares, e tecnologias educacionais não digitais, que proporcionam oportunidades práticas e interativas para o aprendizado sobre diabetes.

**Conclusão:**

Os resultados destacam a relevância das tecnologias educacionais no cuidado de crianças com diabetes tipo 1. Contudo, revelam uma lacuna na avaliação da eficácia dessas intervenções em longo prazo, no que diz respeito à adesão ao tratamento e à melhoria da qualidade de vida. São necessárias pesquisas que avaliem a eficácia dessas tecnologias e o impacto das intervenções educativas.

## INTRODUÇÃO

O Diabetes Mellitus Tipo 1 (DM1) é a segunda doença crônica de maior prevalência na infância, com média de 600 mil casos entre crianças e adolescentes na faixa de 0 a 14 anos de idade. O Brasil é o terceiro país com maior número de crianças e adolescentes com diabetes tipo 1 no mundo, com prevalência de aproximadamente 51 mil casos na faixa-etária de 0 a 14 anos^(1)^.

O DM1 é causado por um processo autoimune onde o sistema imunológico ataca as células beta pancreáticas, resultando na diminuição ou ausência da produção de insulina^(1)^. É considerada uma das doenças crônicas da infância mais comuns e graves, devido às complicações que pode causar em curto, médio e longo prazos, como lesão nos vasos sanguíneos, no coração e cérebro, retinopatia, neuropatia, nefropatia e cetoacidose diabética, dentre as quais a doença renal é a mais frequente e mais precocemente encontrada em jovens com diabetes^(1,2)^.

A doença crônica na infância limita as atividades diárias, reflete no processo de crescimento e desenvolvimento, requer assistência de saúde contínua e afeta o cotidiano de todos os membros da família. Por isso, os familiares que assumem a responsabilidade pelo cuidado precisam adquirir conhecimentos sobre a alimentação, sinais e sintomas de hipoglicemia e hiperglicemia, além de serem treinados para o preparo e administração de insulina^(3)^.

A educação em saúde é um importante alicerce no cuidado da criança com diabetes, promovendo ações para a melhoria e efetividade dos cuidados prestados no ambiente domiciliar às crianças com essa doença crônica^(4)^.

No contexto do DM1, diferentes tecnologias educacionais (TE) podem ser incorporadas para subsidiar as ações educativas dos profissionais de saúde, facilitar o aprendizado e favorecer a adesão ao tratamento e ao autocuidado^(5)^.

Posto isso, realizou-se uma busca preliminar, em julho de 2023, no PROSPERO (*International Prospective Register of Systematic Reviews*) e nas principais bases de dados e nenhuma revisão de escopo ou sistemática, atual ou em andamento, sobre a temática, foi identificada.

Ao considerar a relevância do tema, esta revisão tem como objetivo mapear, por meio de produções científicas, as tecnologias educacionais para familiares e crianças com diabetes mellitus tipo 1.

Espera-se que a presente revisão de escopo forneça subsídios para novos estudos sobre o tema em questão, para favorecer a adesão de familiares e de crianças por meio de tecnologias educacionais no enfrentamento do DM1.

## MÉTODO

### Desenho do Estudo

Trata-se de uma revisão de escopo, conduzida conforme as recomendações do JBI^(6)^ e descrita de acordo com o *checklist Preferred Reporting Items for Systematic Reviews and Meta-Analyses Extension for Scoping Reviews* (PRISMA-ScR)^(7)^. Na fase exploratória desta revisão, objetivamos garantir a ausência de relatório de pesquisa recente semelhante à temática em estudo ou registro de protocolo de revisão. Posteriormente, o protocolo desta revisão foi registrado na plataforma *Open Science Framework* (https://doi.org/10.17605/OSF.IO/A72F4).

### Identificação da Questão de Pesquisa

Revisão conduzida pela seguinte questão: “Quais as tecnologias educacionais para familiares e crianças com diabetes tipo 1? Para a construção da questão de pesquisa, utilizou-se a estratégia mnemônica PCC (População, Conceito e Contexto), considerando como população (P) - crianças com diabetes tipo 1 e/ou familiares; o conceito (C) – as tecnologias educacionais; e o contexto (C) – ambientes de cuidado em saúde.

Foram considerados como critérios de inclusão estudos cuja população era composta por crianças de 2 a 12 anos e adolescentes, e como critérios de exclusão estudos cuja população era exclusivamente composta por adolescentes, estudos que tratam especificamente da cetoacidose diabética e estudos direcionados exclusivamente para professores ou profissionais de saúde.

### Critérios de Seleção

foram considerados estudos experimentais e quase-experimentais (estudos controlados randomizados e não randomizados, estudos tipo antes e depois e estudos de séries temporais interrompidos), estudos observacionais analíticos (estudos de coorte prospectivos e retrospectivos, estudos caso-controle e estudos analíticos transversais), estudos observacionais descritivos (séries de casos, relatos de casos individuais e estudos transversais descritivos), estudos qualitativos. Revisões sistemáticas, que atendiam aos critérios de inclusão, também foram consideradas para análise e seleção de suas listas de referências.

### Estratégia de Busca

A busca foi realizada nas bases de dados LILACS, BDENF, Coleciona SUS, dentre outras do Portal Regional da BVS, MEDLINE via Pubmed/NLM, Pubmed Central/NLM, COCHRANE Library, CINAHL, EBSCO, Scopus e Embase/Elsevier, Web of Science/Clarivate Analytics, Scielo, de acordo com a sintaxe e termos de indexação apropriados para cada base. Da mesma maneira, foram considerados o Google, Google Scholar e as fontes de literatura cinzenta, como por exemplo Science.gov e USA.gov. A estratégia de busca está listada no Quadro 1. As referências de todas as fontes de evidência incluídas foram examinadas na busca de estudos adicionais. Para ampliar os achados, os revisores definiram a estratégia com auxílio de uma bibliotecária.

**Quadro 1:** Estratégia de busca utilizada na revisão – Niterói, RJ, Brasil, 2023.

((“Diabetes Mellitus, Type 1”[mh] OR “Diabetes Mellitus, Type 1”[tiab] OR “Autoimmune Diabetes”[tiab] OR “Brittle Diabetes Mellitus”[tiab] OR IDDM[tiab] OR “Insulin Dependent Diabetes Mellitus 1”[tiab] OR “Insulin-Dependent Diabetes Mellitus”[tiab] OR “Insulin-Dependent Diabetes Mellitus 1”[tiab] OR “Juvenile Onset Diabetes”[tiab] OR “Juvenile-Onset Diabetes”[tiab] OR “Juvenile-Onset Diabetes Mellitus”[tiab] OR “Ketosis-Prone Diabetes Mellitus”[tiab] OR “Sudden-Onset Diabetes Mellitus”[tiab] OR “Type 1 Diabetes”[tiab] OR Type 1 Diabetes Mellitus[tiab]) AND (“Health Education”[mh] OR “Health Education”[tiab] OR Educac*[tiab] OR “Educational Technology”[mh] OR Educational Technolog*[tiab] OR Instructional Technolog*[tiab] OR “health fairs”[tiab] OR health science* education[tiab] OR Pamphlets[mh] OR Booklet*[tiab] OR Brochure*[tiab] OR Pamphlet* Instructional[tiab] OR Instructional Film[tiab] OR Instructional Video[tiab] OR Instruction[tiab] OR Audiovisual Demonstration[tiab] OR Audio-Video Demonstration[tiab] OR Audiovisual Demonstration[tiab] OR Instruction[tiab] OR Instructional Films[tiab] OR Instructional Videos[tiab] OR Video-Audio Demonstration[tiab] OR “Audiovisual Aids”[mh] OR Audio Visual Aid*[tiab] OR Audio-Visual Aid*[tiab] OR Audiovisual Aid[tiab] OR Visual Aid*[tiab] OR “Teaching Materials”[mh] OR “Teaching Material”[tiab] OR booklet[tiab])) AND (“Infant”[mh] OR Infant*[tiab] OR “Child, Preschool”[mh] OR Preschool*[tiab] OR Pediatric*[tiab] OR “Child”[mh] OR Child*[tiab] OR Children[tiab] OR Non-Professional Home Care[tiab] OR Nonprofessional Home Care[tiab] OR Housing[mh] OR Family Patient Lodging[tiab] OR Family-Patient Lodging*[tiab] OR Patient Family Lodging*[tiab] OR Patient-Family Lodging*[tiab] OR “Home Environment”[mh] OR Home Environment*[tiab] OR Living Alone[tiab] OR Social Housing Condition*[tiab] OR “Foster Home Care”[mh] OR Adult Foster Care[tiab] OR Fostering[tiab] OR Kinship Care[tiab] OR “Caregivers”[mh] OR Caregiver*[tiab] OR Carer*[tiab] OR “Care Givers”[tiab] OR “Care Giver”[tiab] OR family care*[tiab] OR “unpaid care”[tiab] OR informal care*[tiab] OR “Family”[mh] OR Families[tiab] OR Filiation[tiab] OR relatives[tiab] OR Stepfamil*[tiab] OR Parent*[tiab] OR “Step Parents”[tiab] OR Step-Parent[tiab] OR Step-Parent*[tiab] OR Stepparent*[tiab] OR maternity[tiab] OR motherhood[tiab] OR parenthood[tiab] OR paternity[tiab] OR “mothers”[mh] OR “Fathers”[mh] OR “mothers”[tiab] OR”Fathers”[tiab])

### Seleção dos Estudos

Para as etapas iniciais de seleção dos estudos, utilizou-se a plataforma Rayyan QCRI® (the Systematic Reviews web app). Dois revisores independentes verificaram a relevância dos estudos selecionados, mediante a leitura dos títulos e resumos. As divergências em relação à inclusão dos artigos foram resolvidas por meio de discussão entre os revisores, sem a necessidade da intervenção de um terceiro revisor. Posteriormente, os artigos selecionados foram lidos na íntegra, o que precedeu sua inclusão na amostra final.

#### Extração e Síntese dos Dados

Para sistematizar os resultados, os dados extraídos foram compilados, de forma descritiva, em um quadro desenvolvido pelos autores e adaptados ao recomendado pelo manual do JBI^(6)^. Foram destacadas as informações: autor, ano, país, tipo de estudo, participantes, tipo e conteúdo das tecnologias.

## RESULTADOS

A busca nas bases de dados ocorreu no período de setembro a dezembro de 2023. A estratégia de busca utilizada resultou em 4375 registros, dos quais, após a remoção de duplicatas, 3296 artigos foram incluídos para a triagem inicial e análise de títulos e resumos. Dessa etapa, 165 artigos foram selecionados para leitura completa. Dentre os selecionados, 86 foram excluídos por não abordarem totalmente o tema e 29 não puderam ser recuperados, totalizando 50 artigos incluídos. Além disso, após a análise das referências, mais 3 estudos foram acrescentados, compondo uma amostra final de 53 estudos selecionados para a revisão (Figura 1).

**Figura 1:** Diagrama de fluxo PRISMA-ScR do processo de seleção das publicações da revisão. Niterói, RJ, Brasil, 2023.

A maioria dos artigos foi publicada entre 2014 e 2020. Em relação à origem dos estudos, o Brasil foi o país com a maior produção científica sobre a temática, contabilizando 17 artigos, seguido pelos Estados Unidos, com 16 artigos. A maior concentração de estudos foi de ensaios experimentais (13 estudos) (Quadro 2).

**Quadro 2:** Caracterização dos estudos incluídos na amostra final da revisão – Niterói, RJ, Brasil, 2023.

Cód	Autor Ano País	Tipo de Estudo	Participantes	Tecnologias Educacionais
A1^(8)^	Sunni M et al. 2023 EUA	Estudo transversal	22 pais somalis de 22 crianças	Vídeos educativos
A2^(9)^	Merino MFGL et al. 2022 Brasil	Estudo qualitativo, convergente-assistencial	16 crianças/ adolescentes	Ações educativas: brinquedos, jogos, vídeos e materiais impressos
A3^(10)^	Ribeiro ALT et al. 2021 Brasil	Estudo metodológico	16 Crianças/ adolescentes	História em quadrinhos
A4^(11)^	Sparapani VC et al. 2021 Brasil	Estudo metodológico	19 crianças	Programa de instrução assistida por computador
A5^(12)^	Araújo et al. 2020 Brasil	Estudo metodológico	16 Crianças/ adolescentes	História em quadrinhos
A6^(13)^	La Banca RO et al. 2020 Brasil	Estudo qualitativo descritivo	20 crianças/ adolescentes	Brinquedo Terapêutico Instrucional
A7^(14)^	Chaves FF et al. 2020 Brasil	Estudo metodológico	10 crianças/ adolescentes	Protocolo para App
A8^(15)^	La Banca RO et al. 2020 Brasil	Ensaio piloto randomizado	20 crianças	Intervenção lúdica terapêutica (PTI)
A9^(16)^	La Banca RO et al. 2019 Brasil	Estudo de caso qualitativo integrado	2 Crianças	Brinquedo terapêutico instrucional
A10^(17)^	Serafim AR et al. 2019 Brasil	Estudo metodológico	16 crianças/ adolescentes	Serious games
A11^(18)^	Albanese-O’Neill A et al. 2019 EUA	Estudo de métodos mistos	30 Pais de crianças com DM1	Site
A12^(19)^	Rafeezadeh E et al. 2019 Irã	Ensaio Clínico Randomizado	68 crianças	Videogame educacional interativo
A13^(20)^	Marker AM et al. 2019 EUA	Estudos experimental	14 pais/mães	Plataforma online: micro-palestras em vídeo
A14^(21)^	Kyfonidis C, Lennon M 2019 Escócia	Estudo experimental	27 pais e 21 crianças	Atividade interativa com brinquedos educativos tangíveis
A15^(22)^	Emiliana P et al. 2019 Indonésia	Estudo quase experimental sem grupo de controle	31 crianças	Vídeos animados
A16^(23)^	Pennafort VPS et al. 2018 Brasil	Estudo qualitativo	26 crianças	Brinquedo terapêutico no cuidado cultural
A17^(24)^	Kaneto LA et al. 2018 Brasil	Estudo quase-experimental	33 crianças	Oficina educativa/jogos
A18^(25)^	Bernier A et al. 2018 EUA	Ensaio Clínico Randomizado	16 crianças	Aplicativo educacional
A19^(26)^	Henkemans OAB et al. 2017 Holanda	Ensaio Clínico Randomizado	27 crianças/ adolescentes	Robô pessoal
A20^(27)^	Moura DJM et al. 2017 Brasil	Pesquisa Metodológica	19 crianças de 8 a 11 anos	Cartilha educativa
A21^(28)^	McCulloch V et al. 2017 Reino Unido	Estudo experimental	5 crianças/ Adolescentes	Aplicativo móvel educativo e interativo
A22^(29)^	Bechara GM et al. 2017 Brasil/Belgica	Estudo experimental	9.944 alunos, destes 26 com DM1; 236 professores e 32 familiares	Programa Kids (pacote informativo online)
A23^(30)^	Mauri A et al. 2017 Itália	Estudo metodológico	24 crianças	Programa de educação terapêutica (oficinas educativas)
A24^(31)^	Nilsson S 2016 Suécia	Estudo qualitativo descritivo	15 crianças	Tablet: app anatomia humana, jogo diabetes, contador de carboidratos.
A25^(32)^	Joubert M et al. 2016 França	Estudo piloto multicêntrico prospectivo	38 crianças	Jogos serious
A26^(33)^	Ramchandani N et al. 2016 EUA	Estudo qualitativo descritivo	31 mães e 18 pais	Simulador de paciente humano
A27^(34)^	Sullivan-Bolyai S et al. 2016 EUA	Ensaio clínico randomizado	22 pais e 22 crianças	Simulador de paciente humano
A28^(35)^	Queiroz MVO et al. 2016 Brasil	Estudo descritivo e analítico	6 crianças	Dinâmica do Conhecimento Corporal e desenhos
A29^(36)^	Chomutare T et al. 2016 Noruega	Design participativo	14 crianças	Co-design ou design participativo de jogos sérios em dispositivos móveis
A30^(37)^	Price KJ et al. 2015 Reino Unido	Ensaio Clínico Randomizado	396 crianças/ adolescentes	Curso de educação em diabetes
A31^(38)^	Maguire LL et al. 2015 EUA	Ensaio Clínico Randomizado	30 avós de crianças com diabetes	Simulador de paciente humano
A32^(39)^	Bin-Abbas B et al. 2014 Arábia Saudita	Ensaio experimental prospectivo	200 crianças	Sistema de mensagens interativas, informativas e multimídia
A33^(40)^	Narae Kang et al. 2014 Coréia	Estudo quase experimental	15 crianças/ adolescentes	Palestra, quiz e jogos
A34^(41)^	Jaser SS et al. 2014 EUA	Ensaio Clínico Multicêntrico	320 crianças/ adolescentes	Programa psicoeducacional online - habilidades de enfrentamento e autocuidado
A35^(42)^	Grey M et al. 2013 EUA	Ensaio Clínico Multicêntrico	320 crianças/ adolescentes	Programa psicoeducacional baseado na internet - vídeo de história em quadrinhos e links com exercícios de resolução de problemas
A36^(43)^	Hanberger L et al. 2013 Suécia	Ensaio clínico randomizado	487 (289 crianças/adolescentes e 198 pais)	Portal web
*A37^(44)^	Ramchandani N et al. 2016 EUA	Estudo qualitativo descritivo	Pais de crianças > 13 anos (Não descreve o quantitativo de participantes)	Simulador de paciente humano
A38^(45)^	Nicholas DB et al. 2012 Canadá	Estudo experimental com métodos mistos	74 crianças/adolescentes	Site
A39^(46)^	Sullivan-Bolyai S et al. 2012 EUA	Um estudo de grupo focal e dois estudos piloto	16 pais	Simulador de paciente humano
A40^(47)^	Fuchslocher A, Niesenhaus J, Krämer N 2011 Alemanhã	Estudo experimental intersujeitos com amostra clínica	20 crianças	Jogo digital
A41^(48)^	Souza LR et al. 2010 Brasil	Estudo qualitativo descritivo	10 crianças e família	Folheto educativo
A42^(49)^	Martin C, Liveley K, Whitehead K 2009 Reino Unido	Estudo experimental	5 crianças	Sessão grupal: contação de história e desenhos após/ Sessão individual: app e jogos
A43^(50)^	Wangberg SC, Årsand E, Andersson N 2006 Noruega	Ensaio Clínico	15 crianças	Sistema de mensagem de texto – SMS
A44^(51)^	Pélicand J et al. 2006 França	Estudo quase-experimental com grupo controle	14 crianças	Programa de educação terapêutica (confecção de fantoches; jogos de cartas; leitura; dramatização.
A45^(52)^	Noriaki Aoki et al. 2005 EUA	Estudo experimental	30 crianças/ adolescentes	App jogo
A46^(53)^	Nordfeldt S et al. 2005 Suécia	Estudo randomizado	332 crianças/ adolescentes	Material de autoestudo em vídeos
A47^(54)^	Noriaki Aoki et al. 2004 Japão	Ensaio Clínico	58 crianças	Videogame
A48^(55)^	Nordfeldt S et al. 2003 Suécia	Estudo randomizado	332 crianças/ adolescentes	Fitas de vídeo e um folheto
A49^(56)^	Nordfeldt S, Ludvigsson J 2002 Suécia	Desenho de coorte aberta	139 crianças/adoles centes	Folhetos de autoestudo e programas de vídeo
A50^(57)^	Brown SJ et al. 1997 EUA	Ensaio Clínico Randomizado	31crianças/ adolescentes	Videogame
A51^(58)^	Engvall JC 1994 EUA	Estudo Descritivo	50 Crianças	Videogame
*A52^(59)^	Rogari GL, Uster M 1988 EUA	Estudo experimental	Crianças com diabetes (Não descreve o quantitativo de participantes)	Programa educativo: palestras, slides, quadro-negro, jogos, dramatização.
A53^(60)^	James VH, Susan JL 1980 EUA	Estudo experimental	37 crianças/ adolescentes	App Jogo

*Os artigos não descrevem o quantitativo de participantes.

### Categorização das Tecnologias Educacionais

As tecnologias educacionais foram organizadas em duas categorias: tecnologias educacionais digitais e não digitais, compreendendo os dados mais relevantes encontrados neste estudo. As TE digitais foram os sites, plataformas, vídeos, videogame, aplicativos, jogos digitais, simuladores de pacientes humanos e mensagens de texto (via celular). As não digitais foram cartilhas, folhetos, livros, história em quadrinhos, brinquedo terapêutico, palestras, quiz, fantoches, oficinas, grupos e programas educativos. Os temas centrais explorados nas TE abordaram insulinoterapia, monitorização glicêmica, hiperglicemia, hipoglicemia, atividade física, alimentação, fisiopatologia do diabetes, complicações tardias, escola e tecnologias para diabetes.

### Tecnologias Educacionais Digitais

Foram encontrados 37 artigos que abordam tecnologias digitais^(8,11,14,17-22,25,26,28,29,31-34,36-39,41-47,50,52-58,60)^.

Com relação aos vídeos^(8,10,22,53,55,56)^, estes proporcionam uma abordagem acessível, com boa aceitação cultural e eficaz na disseminação de informações. Já os videogames^(11,19,32,36,47,54,57)^ apresentam-se como ferramentas com abordagem inovadora para crianças com DM.

Os aplicativos móveis e *serious games* ^(14,17,25,28,31,52)^ são úteis ao oferecer recursos como mensagens de apoio, monitoramento glicêmico e registro emocional, propiciando treino de habilidades operacionais e comportamentais do jogador.

Alguns estudos destacam o potencial das tecnologias móveis, como mensagens de celular^(18,39,50)^ e programas educativos telemédicos^(43,45)^, para oferecer educação em diabetes acessível e fora do ambiente clínico. Destacou-se ainda o uso de simuladores de pacientes humanos^(21,26,33,34,38,44,46)^, materiais educativos domiciliares e programas de ensino assistidos por computador^(29,37,41,42,53,55,56,58)^.

### Tecnologias Educacionais Não Digitais

Nesta categoria, foram incluídos 18 artigos^(9,10,12,13,15,16,23,24,27,30,35,40,48,49,51,55,56,59)^ que abordam tecnologias não digitais. Dentre esses, 3 estudos^(9,55,56)^ adotaram abordagens que combinam tanto tecnologias digitais quanto não digitais para alcançar seus objetivos educacionais.

Foram utilizados brinquedo terapêutico^(13,15,16,21,23)^, materiais impressos (cartilhas, folhetos, livros, história em quadrinhos)^(10,12,27,48,55,56)^, programas educativos implementados tanto em acampamentos especializados em diabetes^(40,49,51)^ quanto em um ambiente de ambulatório hospitalar^(59)^, nos quais uma variedade de atividades foi conduzida, incluindo contação de histórias, uso de fantoches, jogos, dramatizações, palestras e quizzes. Também foram propostas dinâmicas e oficinas^(24,35)^, além de estratégias específicas para abordar cada dificuldade relatada tanto pela criança quanto pela mãe, com atividades personalizadas adaptadas ao nível de compreensão de cada díade^(9)^.

## DISCUSSÃO

As TEs digitais desempenham um papel crucial no manejo do DM1, fornecendo recursos culturalmente sensíveis e personalizados para educar pacientes e suas famílias. Isso inclui o uso de videogames, aplicativos móveis, simuladores e intervenções online, que visam promover o conhecimento sobre o DM1, estimular o autocuidado e melhorar a qualidade de vida dos pacientes. O uso de recursos tecnológicos, mais atraentes e voltados à realidade, pode facilitar a capacitação, estimular o raciocínio e a capacidade de resolver problemas^(61)^.

Os vídeos, citados por 5 estudos desta revisão, são tecnologias acessíveis, que podem ser editados e adaptados a diversidades culturais, são eficazes na disseminação de informações. Eles podem ser traduzidos em diferentes idiomas, dublados ou legendados, e assim utilizados junto a diversos públicos, e ainda facilmente distribuídos aos pacientes mediante sites, mídias sociais, mensagem de texto. Também pode haver inclusão de intérpretes de LIBRAS e assim incluir o público deficiente auditivo. Os vídeos educativos são uma estratégia eficaz, pois captam a atenção do público, estimulam a criatividade e despertam a curiosidade sobre o tema, facilitando a prática educativa de forma direta e simples^(62)^.

Os videogames, que também foram tecnologias encontradas na revisão, apresentam-se como ferramentas educacionais inovadoras. Esses jogos são desenvolvidos com base em teorias comportamentais e incentivam a participação ativa das crianças, promovendo o conhecimento sobre a doença e comportamentos de autocuidado de maneira lúdica. Os videogames proporcionam benefícios cognitivos, como foco de atenção, habilidades de resolução de problemas e estímulo à criatividade. Eles também têm repercussões positivas na motivação, humor e sentimentos positivos. Ademais, favorecem comportamentos sociais, como cooperação e compromisso^(63)^.

Os aplicativos móveis foram apontados como importantes tecnologias educacionais, pois oferecem recursos como mensagens de apoio, monitoramento glicêmico e registro emocional. Dessa forma, complementam o autogerenciamento do DM1 em crianças e adolescentes, numa combinação de informações educativas e ferramentas práticas para controlar a doença de maneira mais eficiente. Esses achados ressaltam a eficácia das tecnologias móveis na capacitação dos pais e no apoio ao cuidado das crianças com DM1, incentivando o autocuidado, melhor controle glicêmico, prática de atividade física, adoção de alimentação saudável e manutenção da medicação prescrita^(64)^.

As tecnologias móveis, como mensagens de celular e programas educativos de telemedicina mostraram-se capazes de oferecer educação em diabetes acessível e fora do ambiente clínico. Estudos indicam que a teleconsulta e o teleatendimento viabilizam o acompanhamento de pacientes pediátricos, possibilitando a avaliação e monitoramento em seus ambientes habituais, como domicílio, escola e comunidade. Essa abordagem melhora o acesso e a qualidade dos cuidados de saúde^(65)^.

Outra tecnologia educacional descrita na literatura são os simuladores de pacientes humanos (HPS). Esses simuladores são dispositivos tecnológicos projetados para imitar situações clínicas reais, permitindo que os cuidadores pratiquem habilidades de manejo do DM1 em um ambiente simulado. A simulação realística tem se destacado como uma abordagem mais efetiva no ensino e aprendizagem do que o ensino tradicional. Essa estratégia proporciona uma experiência prática mais próxima da realidade, contribuindo para uma assistência mais segura e livre de riscos^(66)^.

Novas tecnologias, como o uso da internet para compartilhamento de materiais educativos, comunicações online para o DM1, programas assistidos por computador, emergiram na revisão como ferramentas promissoras para melhorar o cuidado. Neste sentido, estudos sobre plataformas de saúde online e gestão de doenças crônicas indicam que as interações virtuais contribuem para o maior conhecimento da doença pelo paciente, por meio do compartilhamento de experiências e informações; melhor autogestão da condição crônica, devido à troca de experiências e aconselhamento; e maior suporte social, incluindo reforço positivo e compartilhamento de informações e experiências^(67)^.

Com relação às tecnologias não digitais, o brinquedo terapêutico voltado para população infantil que vive com DM1 emergiu como uma intervenção lúdico-terapêutica promotora de troca de experiências entre a criança e o profissional de saúde, bem como entre os pares durante atividades em grupo. Como benefícios, ele amplia o conhecimento sobre a doença e facilita o treinamento prático de habilidades essenciais para o cuidado do diabetes, tais como monitorização glicêmica e aplicação de insulina. Os resultados obtidos nesta revisão mostram eficácia na promoção do autocuidado por parte das crianças.

Os jogos e brinquedos desempenham papel terapêutico significativo, incentivando a criança a expressar seus sentimentos e ansiedades em relação à sua condição de saúde, ajudando-a a aliviar a tensão imposta pela sua realidade. Além disso, essas atividades promovem uma maior interação criança-profissional^(68)^.

Os diversos tipos de materiais educativos impressos, encontrados nos resultados da revisão, são utilizados como recursos para autoestudo e complementam as orientações realizadas pelos profissionais de saúde. Conforme estudos, os materiais impressos, como a cartilha, são recursos que permanecem disponíveis para consulta por parte do usuário e sua família diante de dúvidas, facilitando o processo de autonomia, e uma maior adesão ao tratamento e compreensão sobre a influência de suas próprias ações no padrão de saúde^(69)^.

Programas educativos, com atividades lúdicas e palestras, desenvolvidos em acampamentos e ambulatórios foram apontados como possibilidades de atuação junto à população de estudo. Esses métodos interativos e recreativos proporcionam às crianças uma oportunidade de expressar e compartilhar suas dificuldades relacionadas ao diabetes, corroborando ainda para as habilidades psicossociais. Conforme evidenciado em uma revisão sistemática sobre estratégias educativas utilizadas no ensino da insulinoterapia às crianças e adolescentes com DM1, os programas educativos devem priorizar não apenas estratégias que levem em conta as necessidades e particularidades da população-alvo, mas também garantir um apoio contínuo. Isso é fundamental para o desenvolvimento e a manutenção de comportamentos de autocuidado, alcançando melhores resultados ao longo do tempo^(70)^.

Os temas centrais explorados nas TE apresentadas nesta revisão mostram a necessidade de uma abordagem integrada que englobe não apenas a terapia com insulina e monitoramento glicêmico, mas também a nutrição, a atividade física e as habilidades de enfrentamento. São temas consistentes com as recomendações da *American Diabetes Association* (ADA), que enfatiza a importância da educação abrangente e contínua para otimizar os resultados de saúde em pacientes com diabetes^(2)^, fornecendo uma base sólida para orientar eficazmente as crianças com DM1 e seus familiares no autocuidado e na promoção de uma melhor qualidade de vida.

Esta revisão identificou como potencial limitação a exclusão de 29 artigos não recuperados na íntegra, o que pode gerar um viés na revisão, pois esses estudos poderiam conter dados relevantes que não foram considerados.

No que concerne às implicações para a prática da enfermagem, esta revisão proporciona uma síntese das tecnologias educacionais e os temas abordados no cuidado da criança com diabetes descritas na literatura, as quais podem ser replicadas, adaptadas e empregadas pelos enfermeiros em suas intervenções de educação em saúde voltadas para crianças com diabetes e seus familiares. Essa análise oferece suporte para o manejo eficaz do diabetes e a promoção da melhoria da qualidade de vida desses pacientes e suas famílias.

## CONCLUSÃO

Esta revisão de escopo destacou a diversidade e a importância das tecnologias educacionais no manejo do DM1 em crianças, revelando uma gama de ferramentas digitais e não digitais utilizadas para promover o autocuidado e apoiar as famílias. Os estudos analisados indicaram que essas tecnologias não apenas aumentam o conhecimento sobre a doença, mas também contribuem para o desenvolvimento de habilidades práticas e comportamentais necessárias para o manejo efetivo do DM1.

Entretanto, foram identificadas várias lacunas na literatura. Muitos estudos concentraram-se principalmente na descrição do desenvolvimento das tecnologias educacionais, sem aprofundar na análise dos conteúdos temáticos abordados. Além disso, a disseminação científica dessas tecnologias ainda é limitada, restringindo seu acesso a um público mais amplo. Também se observou uma carência de estudos que avaliem a efetividade dessas tecnologias em longo prazo e seu impacto na adesão ao tratamento e na qualidade de vida das crianças e suas famílias.

Portanto, há uma necessidade clara de estudos futuros que avaliem a eficácia das tecnologias educacionais ao longo do tempo, considerando diferentes contextos culturais e socioeconômicos; investiguem a implementação dessas tecnologias em larga escala, visando sua integração nos sistemas de saúde pública; e explore o conteúdo temático das intervenções educacionais com maior profundidade, fornecendo diretrizes específicas para os profissionais de saúde.

Essas investigações futuras podem ajudar a consolidar as evidências existentes e a expandir o uso das tecnologias educacionais, promovendo um cuidado mais eficaz e abrangente para crianças com diabetes tipo 1 e suas famílias.
